# Minimally Invasive Approach Versus Traditional Approach for Treating Congenital Diaphragmatic Hernia: A Systematic Review and Meta-Analysis

**DOI:** 10.7759/cureus.77596

**Published:** 2025-01-17

**Authors:** Abdulkreem Aljuhani, Ahmed A Alsumaili, Eman M Alyaseen, Lojain I Daak, Abdullah Esmail, Jood E Alzohari, Abdullah Alqahtani, Eyesha A Junaidallah, Hashem A Alghamdi, Fajr Saeedi

**Affiliations:** 1 General Surgery, King Abdulaziz University, Faculty of Medicine, Jeddah, SAU; 2 College of Medicine, King Abdulaziz University, Faculty of Medicine, Jeddah, SAU; 3 College of Medicine and Medical Science, Arabian Gulf University, Manama, BHR; 4 College of Medicine, Jazan University, Jazan, SAU; 5 Clinical Sciences, Al Rajhi University, Qassim, SAU; 6 Pediatric Surgery, Jeddah University, Jeddah, SAU; 7 College of Medicine, King Saud bin Abdulaziz University for Health Science, Riyadh, SAU; 8 Pediatric Surgery, Al Maarefa University, Riyadh, SAU; 9 Plastic and Reconstructive Surgery, King Khalid University, Abha, SAU; 10 Pediatrics, King Abdulaziz University, Faculty of Medicine, Jeddah, SAU

**Keywords:** cdh, congenital diaphragmatic hernia, open repair, pediatric surgery, thoracoscopic repair

## Abstract

Congenital diaphragmatic hernia (CDH) is a rare but critical surgical disorder that can be managed using either open or thoracoscopic surgical approaches. However, the optimal approach remains a topic of debate. This study aimed to evaluate the efficacy and safety of thoracoscopic repair compared to open repair in patients with CDH. An extensive literature search was conducted across four databases (PubMed, Web of Science, Scopus, and Cochrane Library) from inception to May 2024, including all relevant studies comparing the two surgical modalities. Key outcomes assessed were hospital stay duration, operation time, mortality, and recurrence. Categorical outcomes were analyzed using the risk ratio (RR) with 95% confidence intervals (CI), while continuous outcomes were analyzed using the mean difference (MD) with 95% CI. Data analysis was performed using Review Manager (RevMan, Version 5.3).

A total of 35 studies involving 1,680 individuals with CDH were included in our analysis. The pooled results revealed that thoracoscopic repair was associated with a shorter hospital stay (MD=-6.80, 95% CI [-9.39, -4.21], p< 0.0001) but a longer operation time (MD=23.30, 95% CI [7.22, 39.38], p=0.005) compared to the open approach. Additionally, thoracoscopic repair demonstrated lower mortality rates (RR=0.43, 95% CI [0.24, 0.76], p=0.004) but higher recurrence rates (RR=2.24, 95% CI [1.56, 3.21], p<0.0001) than open repair.

Our findings suggest that thoracoscopic repair offers shorter hospital stays and lower mortality rates but involves longer operation times and higher recurrence rates compared to the open approach. These results highlight the need for further large, multicenter, randomized controlled trials to validate our findings and guide clinical decision-making.

## Introduction and background

Congenital diaphragmatic hernia (CDH) results from inadequate closure of the diaphragm during embryonic development, especially within the pivotal period of five to ten weeks post-fertilization [1]. CDH is a life-threatening anomaly affecting approximately two to three per 10000 live births [2]. The defect can exhibit unilateral or bilateral patterns, along with partial or complete manifestations. Elevated intra-abdominal pressure drives the abdominal organs, encompassing the intestine, liver, and spleen, to protrude into the thoracic cavity through the opening, eventually resulting in compression of the adjacent lung parenchyma [1]. The incidence of left-sided CDH exceeds right-sided or bilateral presentations, which are notably rare.

Diaphragmatic hernia can induce diverse impacts on the functions of the gastrointestinal tract, cardiorespiratory system, skeletal musculature, and neurocognition [3]. Right-sided CDH commonly involves the herniation of the liver primarily, which has an echogenicity similar to that of lung tissue, thus raising the probability of diagnostic omission. The protrusion of the abdominal contents into the thoracic cavity during prenatal development disrupts lung maturation in neonates, resulting in postnatal lung dysplasia marked by diminished pulmonary surfactant levels, which eventually results in life-threatening pulmonary hypertension in severe cases [4,5]. Nowadays, surgery stands as the sole definitive treatment for CDH, encompassing open and thoracoscopic procedures as available treatment modalities [6]. Traditionally, the standard technique for managing CDH is open surgical repair, executed through either thoracotomy or laparotomy. Despite its favorable outcomes, the open approach is burdened by extensive tissue trauma, long hospital stays, and heightened rates of complications.

Over recent years, thoracoscopic repair, a minimally invasive surgical intervention, has emerged as an alternative modality for open repair for managing CDH. In contrast, to open repair, the thoracoscopic repair for CDH demonstrates advantages, involving better visual clarity, reduced tissue damage, and procedural efficiency, thus leading to its growing use in clinical settings [7,8]. Furthermore, thoracoscopic repair was linked to a shorter hospital stay, shorter duration of postoperative mechanical ventilation, and early return to feeding compared to open repair [9,10].

Nevertheless, in neonates, the presence of pulmonary hypertension and concurrent congenital anomalies could impose limitations on the applicability of minimally invasive modalities [11]. Additionally, the complicating factors, encompassing the intraoperative pressure effect of artificial pneumothorax pressure on lung tissue during the thoracoscopic procedure, coupled with the necessity for partial lung collapse on the surgical side to maintain optimal visualization for the surgeon, impose notable challenges for anesthesia management during surgery [12]. Owing to these notable differences and challenges between the two procedures, a consensus regarding the optimal surgical approach between the two options remains inconclusive. Based on our recently updated knowledge, our study represents the first systematic review and meta-analysis comparing thoracoscopic and open repair approaches in managing children with CDH. We aimed to update the existing literature with the most updated evidence regarding the difference between the two procedures.

## Review

Methods

Our study was executed in strict adherence to the Cochrane Handbook rules [13]. Adherence to The PRISMA statement guidelines was maintained throughout the reporting of this study [14].

Inclusion Criteria

We included all the eligible studies, with no restriction to specific study design, comparing thoracoscopic and open repair approaches in children with CDH. Our study outcomes were duration of hospital stay, operation time, intensive care unit (ICU) stay, postoperative mechanical ventilation, intraoperative pH, intraoperative PCO_2_, mortality, recurrence, pleural effusion, pneumothorax, and intestinal injury. We excluded review articles, conference abstracts, single-arm studies, and unpublished studies.

Databases Search and Screening 

PubMed, Scopus, Web of Science, and Cochrane Library were systematically searched from inception until May 2024 with no publication date or specific language restriction. Our search strategy was as follows: (Open AND Thoracoscopic AND ("Congenital Diaphragmatic Hernia" OR "Congenital Diaphragmatic Hernias" OR CDH OR "Congenital Diaphragmatic Defect" OR "Congenital Diaphragmatic Defects")). 

Retrieved articles from the literature search were screened in two steps. First, the title and abstract of the gathered articles were evaluated for eligibility using Rayyan [15]. Then, the full text of eligible articles was screened and reviewed using Google Sheets. Duplicate articles were eliminated utilizing Endnote (Clarivate Analytics, PA, USA).

Data Extraction

A pre-designed data extraction sheet was used for data collection. We extracted the summary features of the eligible studies, involving study design, country, participants number, follow-up duration, and conclusion, and the baseline features of the study population, encompassing gestational age, gender male, age at repair, and weight.

Bias Assessment 

New-Castle-Ottawa Scale (NOS) was employed to appraise the quality of observational studies [16]. NOS assesses the quality of three main items: patient selection, comparability between the two cohorts, and outcome assessment. The studies were evaluated as having good, moderate, or poor quality based on the cumulative score. Cochrane Risk of Bias tool two (ROB2) was used to assess the quality of the randomized controlled trials (RCTs) [17]. ROB2 accesses the risk of bias through these main domains: randomization process, deviation from the intended interventions, missing outcome data, measurement of the outcome, selection of the reported results, and other potential sources of bias. ROB2 evaluates the studies as having low, some concerns or high risk of bias.

Statistical Analysis and Publication Bias

Continuous outcomes were gathered as mean difference (MD) with its related 95 % confidence interval (CI). Categorical outcomes were pooled as risk ratio (RR) with a 95% CI. We employed a random-effect meta-analysis model while pooling the data. A p-value less than 0.05 was considered significant. Significant heterogeneity was considered when the chi-square p-value was less than 0.1 or the I-square was more than 50%. All the data analysis was executed using RevMan (V5.3) for Windows. Also, we generated a funnel plot to outline the association between effect size and standard error and evaluate any potential publication bias.

Results

Search Results

**Figure 1:**
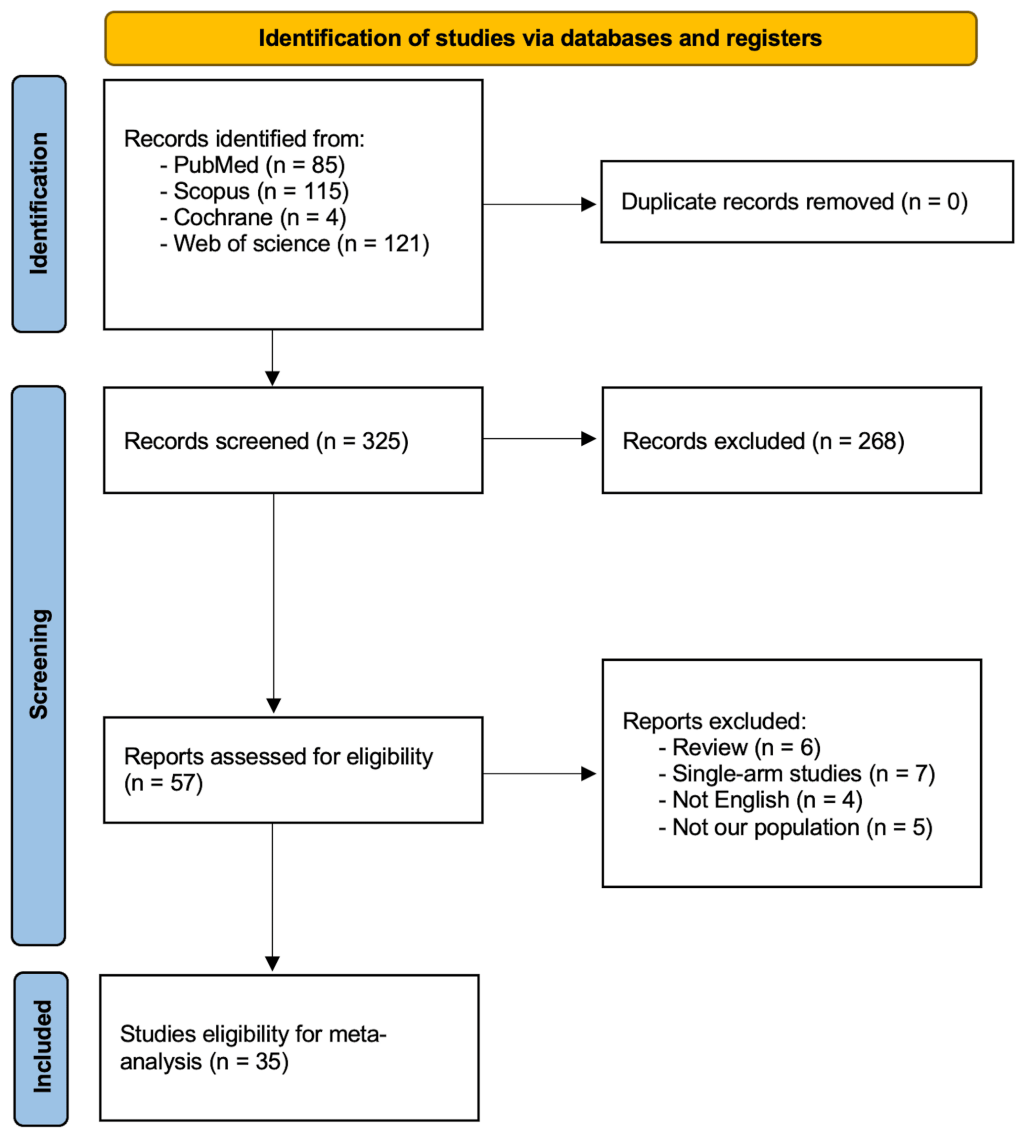
PRISMA 2020 flow diagram for new systematic reviews which included searches of databases and registers only.

Our primary search retrieved 325 records. 269 articles were filtered through the initial screening phase. 57 studies were eligible for the full-text screening. 22 articles were removed after the full-text screening as they did not satisfy our inclusion criteria. Finally, 35 articles were included in our investigation [9,10,12,18-49]. Figure 1 exhibits the PRISMA flow diagram of the study selection process.

Studies Characteristics

Our study finally encompassed 35 studies comparing thoracoscopic versus open procedures for managing children with CDH, with 644 individuals in the thoracoscopic group and 1036 patients in the open group [9,10,12,18-49]. The study design of the retrieved studies was mainly observational (34 studies) and only one RCT. The included studies were conducted in countries such as the USA, Australia, the United Kingdom, Japan, China, and the Netherlands. The mean follow-up period in our study was 26 months. Furthermore, the mean gestational age in our study ranged from 35.5 to 63.95 weeks. The summary and baseline features of the included studies are presented in Table 1 and Table 2.

**Table 1:** Summary features of the included studies. CDH: congenital diaphragmatic defect.

Study ID	Study design	Country	Recruitment time	Recurrent or primary	Number of total participants	Number of thoracoscopic group participants	Number of open group participants	Follow-up period (Months)	Conversion to open surgery, N (%)	Conclusion
Al-iede 2015 [18]	Observational cohort	Australia	Between January 2000 and January 2013	Primary	85	14	71	15	1 (7%)	"A high survival rate of 85% with a 13% incidence of symptomatic CDH recurrence was demonstrated. Potentially, improved selection of cases for thoracoscopic repair and concentration of the thoracoscopic technique amongst a dedicated team of experienced thoracoscopic surgeons may reduce the rate of recurrence of CDH."
Bawazir 2021 [19]	Observational cohort	Saudi Arabia	Between 2011 and 2019	Primary	41	11	30	12	-	"The thoracoscopic approach is safe and effective for repairing the CDH. It is associated with shorter mechanical ventilation and rapid return to enteral feeding. Proper patient selection is essential to achieve good outcomes."
Bishay 2013 [20]	RCT	United Kingdom	-	Primary	10	5	5	14	0 (0%)	"This pilot randomized controlled trial shows that thoracoscopic repair of CDH is associated with prolonged and severe intraoperative by hypercapnia and acidosis, compared with open surgery. These findings do not support the use of thoracoscopy with CO2 insufflation and conventional, even utilization for the repair of CDH, calling into question the safety of this practice."
Budzanowsk 2023 [22]	Observational cohort	United Kingdom	Between 2015 and 2021	Primary	11	6	5	4	-	"Whilst this is a preliminary report of a limited number of patients, there is no obvious difference of intra-operative blood gas parameters during surgical repair in patients after ECMO. Thoracoscopic CDH repair may be considered in patients after ECMO."
Chou 2008 [32]	Observational cohort	USA	Between October 2004 and November 2007	Primary	57	29	28	9.5	1 (3.5%)	"To our knowledge, this is the largest reported series of CDH-T of neonatal CDH in Bochdalek. We have demonstrated the feasibility of performing this procedure thoracoscopically in an unselected population, including children who have undergone prior extracorporeal life support. These results compare favorably with CDH-O, although further follow-up is required to determine the durability of the approach."
Costerus 2015 [21]	Observational cohort	Netherlands and Germany	Between 2008 and 2012	Primary	109	75	34	12	15 (20%)	"After critical selection for thoracoscopic repair of left-sided CDH based on the patient’s preoperative condition, the outcomes of open repair were almost identical to those of thoracoscopic repair. A notable exception is the recurrence rate, which was significantly higher in the thoracoscopic-repair group. For the time being, thoracoscopic primary closure seems a safe and effective procedure, but the efficacy of the thoracoscopic patch repair has not been established."
Criss 2017 [10]	Observational cohort	USA	Between 2006 and 2016	Primary	51	35	16	38	-	"In low-risk patients born with small to moderate-size defects, a thoracoscopic approach was associated with decreased hospital length of stay, mechanical ventilation days, and time to feeding; however, there was a trend towards higher recurrence rates."
Fallahi 2017 [23]	Observational cohort	Iran	Between 2008 and 2015	Primary	74	7	67	11	1 (14%)	"The overall mortality rate in CDH was high in our series. Neonates with CDH should be delivered in institutes with the neonatal intensive care unit and surgery ward to prevent complications."
Fishman 2011 [24]	Observational cohort	United Kingdom	-	Primary	21	12	9	11.6	3 (25%)	"We present our early experience of thoracoscopic CDH repair. Our results from thoracoscopic repair appear similar to the original procedure performed over the same period.”
Gander 2011 [25]	Observational cohort	USA	Between January 2006 and February 2010	Primary	45	26	19	14	-	" Early recurrence of hernia is higher in thoracoscopic CDH repairs than in open repairs. Technical factors and a steep learning curve for thoracoscopy may account for the higher recurrence rates but not the patient severity of illness. In an already-tenuous patient population, performing the repair thoracoscopically with a higher risk of recurrence may not be advantageous."
Gohda 2023 [26]	Observational cohort	Japan	Between January 2003 and October 2022	Recurrent	15	8	7	67	-	"Thoracoscopic surgery is preferable to the open surgical approach for recurrent CDH following an initial abdominal open repair."
Gourlay 2009 [27]	Observational cohort	USA	Between 2004 and 2007	Primary	38	20	18	14.5	-	"Successful thoracoscopic CDH repair can be expected in newborns, which has limited respiratory compromise. Thoracoscopic CDH repair is associated with lower morbidity and quicker recovery than traditional open repair and without increased risk of recurrence or complications."
He 2016 [28]	Observational cohort	China	Between September 2013 and August 2014	Primary	28	14	14	12	1 (7%)	" With selection criteria and timing, TR of CDH in neonates can be performed safely and successfully."
Hendrikx 2022 [29]	Observational cohort	Multicenter (Netherlands-Belgium- Germany-Colombia	Between July 2018 and April 2020	Primary	28	3	25	NA	8 (72%)	"Neurocardiovascular graphs provided more insight into the effect of peri-operative management on the pathophysiology of neonates undergoing surgery. The neonate’s clinical condition, as well as the surgical and the anesthesiologic approach affected the neonatal physiology and CBF regulation mechanisms at different levels."
Hosokawa 2019 [30]	Observational cohort	Japan	Between May 2010 to August 2018	Primary	7	4	3	NA	1 (20%)	"Postnatal US examinations of neonates with CDH could provide surgeons with useful information to determine the surgical approach and repair method. However, since our study cohort was small, further studies are needed with a larger number of neonates with CDH."
Keizer 2010 [31]	Observational cohort	Netherlands	Between June 2006 and December 2008	Primary	46	23	23	12	6 (26%)	"As in open repair, it seems wise to use large patches liberally, not only to reconstruct the dome of the diaphragm but also to avoid undue tension on the repair and prevent recurrences. The thoracoscopic approach is also considered feasible in case of a recurrence from either a thoracoscopic or open repair."
Kim 2009 [33]	Observational cohort	USA	Between 2004 and 2008	Primary	15	12	3	15	3 (20%)	"Thoracoscopic repair of CDH is a safe, effective strategy in patients who have undergone prior stabilization. Stomach herniation is associated with, but does not categorically predict, conversion to open repair. ECMO use prior to repair should not be an absolute contraindication to thoracoscopic repair."
Kunisaki 2012 [34]	Observational cohort	USA	Between January 1990 and March 2011	Recurrent	22	4	18	71	2 (33%)	"Our initial experience suggests that thoracoscopic repair is a feasible alternative to open repair in selected children with recurrent Bochdalek diaphragmatic hernias."
lao 2010 [35]	Observational cohort	USA	Between February 2004 and January 2008	Primary	31	14	17	16	3 (21%)	"In our early experience, the thoracoscopic approach for congenital diaphragmatic hernia repair was completed in 80% of our patient population with minimal exclusion criteria. Further study with larger sample sizes is needed to ascertain differences in outcomes, such as length of stay and initiation of enteral feeding."
Li 2022 [36]	Observational cohort	China	Between January 2011 and January 2021	Primary	109	47	62	54	3 (6%)	"Thoracoscopic surgery and open surgery can effectively treat CDH. Compared with conventional open surgery, thoracoscopy has the advantages of shorter operation time, less trauma, faster recovery, and fewer complications. We believe that thoracoscopic surgery for type A/B diaphragmatic defect has certain advantages, but there is a risk of recurrence."
Lian 2024 [37]	Observational cohort	China	Between July 2002 and November 2021	Primary	64	35	29	114	5 (14%)	"This study is a large congenital diaphragmatic hernia series in Asia, with long-term follow-up demonstrating no significant difference in recurrence rate, time to recurrence, or median length of ICU stay between open and minimally invasive repair, suggesting the thoracoscopic approach is a non-inferior surgical option with avoidance of gastrointestinal complications compared to open repair."
Liu 2022 [9]	Observational cohort	China	Between 2013 and 2021	Primary	50	37	13	23	1 (2%)	"Thoracoscopy CDH repair, a safe and effective surgical technique for neonates, has better cosmesis, faster postoperative recovery, and a lower recurrence rate than other procedures. It can be considered the first choice for CDH treatment for neonates among experienced surgeons."
Mchoney 2010 [38]	Observational cohort	United Kingdom	Between 2003 and 2008	Primary	48	13	35	23	5 (38%)	"Thoracoscopic repair of CDH is feasible. Arterial blood gases should be closely monitored. Despite higher EtCO2, conversion to open was mainly because of difficult repair. A randomized trial is necessary to assess the effect of thoracoscopy on ventilation and recurrences."
Muensterer 2023 [39]	Obervational cohort	NA	Between February 2021 and October 2023	Primary	13	5	8	16	-	"Barbed sutures simplify congenital diaphragmatic hernia repair regardless of whether a minimal-invasive or open approach is performed. Patch repair is not a contraindication for using barbed sutures. The resulting potential time savings make them particularly useful in patients with cardiac or other severe co-morbidities in which shorter operative times are essential. In high-tension cases, though, the barbs may tear through and produce a “saw” effect on the tissue with subsequent damage."
Okawada 2021 [40]	Observational cohort	Japan	Between 2006 and 2018	Primary	228	38	190	6	-	"TR can be performed safely for selected CDH neonates with potentially better outcomes than OR.."
Okazaki 2010 [41]	Observational cohort	Japan	Between January 2007 and December 2009	Primary	21	8	13	NA	3 (37%)	"Our selection criteria for TR would appear to be safe and reasonable."
Okazaki 2012 [42]	Observational cohort	Japan	Between January 2007 and December 2011	Primary	24	10	14	NA	4 (40%)	"TR can be considered when RPA/LPA diameters are larger than 3.0/2.5 mm, respectively, and cardiopulmonary status is stable without NO."
Okazaki 2015 [43]	Observational cohort	Japan	Between 2002 and 2014	Primary	25	15	10	33	-	"It would appear that neonatal cases of CDH not requiring NO can tolerate TR despite the transient reversible deterioration in acid/base balance, indicating that TR is safe for the treatment of selected cases of CDH."
Qin 2019 [12]	Observational Case-control	China	Between 2015 and 2018	Primary	70	26	44	NA	-	"The intraoperative hemodynamic parameters of CDH repair under thoracoscopy were more stable, the duration of postoperative mechanical ventilation, antibiotic use, and hospitalization were shortened, and the therapeutic effect was better."
Romnek 2020 [44]	Observational cohort	USA	Between 2012 and 2016	Primary	28	8	20	NA	-	"Although several factors may impact the hospital course of neonates with CDH, we found that patients had a more than 100-fold difference in median opioid consumption following repair with MIS versus an open approach. The study also noted significant variation in analgesic regimens, suggesting other avenues for improved care of postsurgical neonates."
Schlager 2018 [45]	Observational cohort	USA	Between 2001 and 2015	Primary	40	6	34	24	15 (71%)	"Thoracoscopic CDH repair is both safe and feasible after ECMO with no increase in operative morbidity or mortality. Insufflation pressures of 3–7 mmHg is well tolerated without undue increase in end-tidal CO2. When compared to conversion cases, the thoracoscopic repair is associated with significantly decreased time to extubation with no difference in a recurrence."
Shah 2023 [46]	Observational cohort	USA	Between January 2017 and December 2021	Primary	29	16	13	6	3 (15%)	"Although we demonstrate higher disease severity of patients undergoing open repair, thoracoscopic patch repair for Type B/C defects is safe and effective in patients with favorable physiologic status, alleviating concerns for intraoperative acidosis, operative length, and risk of recurrence."
Tanaka 2013 [47]	Observational cohort	Japan	Between 2002 and 2012	Primary	24	10	14	NA	4 (40%)	"Thoracoscopic repair appears to be as effective as OR for treating selected cases of CDH in neonates, with excellent wound cosmesis."
Tyson 2017 [48]	Observational cohort	USA	Between January 2007 to August 2015	Primary	54	25	29	27	10 (28%)	"In our experience, thoracoscopic CDH repair was performed safely and with similar outcomes compared to open repair. In addition to improved cosmesis, thoracoscopic repair may avoid some of the long-term complications of laparotomy. In our series, none of the thoracoscopic CDH repairs recurred. We concluded that thoracoscopic CDH repair is a safe and appropriate technique for select neonates."
Zani 2017 [49]	Observational cohort	United Kingdom	Between 2004 and 2014	Primary	119	23	96	36	8 (23%)	"Neonates undergoing operative repair of congenital diaphragmatic hernia and esophageal atresia/tracheoesophageal fistula develop intraoperative acidosis and hypercapnia, regardless of the approach used. However, this phenomenon is more severe during thoracoscopic repair. Novel modalities to reduce intraoperative gas derangements, particularly during thoracoscopic repair, need to be established."

**Table 2:** Baseline characteristics of the included studies.

Study ID	Study arms	Gestational age (Weeks), Mean (SD)	Age at surgery (Days), Mean (SD)	Male, N (%)	Weight (kg), Mean (SD)	Left defect, N (%)	Right defect, N (%)	Nitric oxide inhalation, N (%)	APGAR at 1min, Mean (SD)	APGAR at 5min, Mean (SD)
Al-iede 2015 [18]	Thoracoscopic	37.9 (2.5)	4 (2.8)	50 (58%)	3 (0.65)	77 (90.5%)	8 (9.5%)	25 (29%)	5.7 (1.9)	6.9 (1.3)
	Open									
Bawazir 2021 [19]	Thoracoscopic	38 (0.58)	3 (0)	7 (63.6%)	2.8 (0.08)	10 (90.9%)	-	2 (18.1%)	-	-
	Open	38 (0.75)	4(0.5)	19 (63.3%)	2.9 (0.18)	28 (93.3%)	-	11 (36.67%)	-	-
Bishay 2013 [20]	Thoracoscopic	39.25 (1.45)	3.75 (1.45)	4 (80%)	3.3 (0.43)	-	-	-	-	-
	Open	38.25 (1.45)	9.25 (7.07)	3 (60%)	3.05 (0.23)	-	-	-	-	-
Budzanowsk 2023 [22]	Thoracoscopic	38.25 (0.8)	20.25 (4.33)	2 (33%)	3.79 (0.24)	5 (83%)	1 (16%)	-	-	-
	Open	38 (0.5)	19.25 (4.35)	4 (80%)	3.29 (0.21)	3 (60%)	2 (40%)	-	-	-
Chou 2008 [32]	Thoracoscopic	38.1 (2.7)	-	15 (51%)	3.2 (0.5)	21 (72.4%)	-	-	5 (3.1)	-
	Open	38.3 (1.6)	-	16 (57%)	3.1 (0.5)	23 (82.1%)	-	-	4.3 (2.3)	-
Costerus 2015 [21]	Thoracoscopic	38.25 (0.50)	-	41 (54%)	3 (0.3)	-	-	-	6.83 (1.89)	8.33 (0.8)
	Open	37.76 (1.20)	-	16 (47%)	2.8 (0.4)	-	-	-	6.33 (1.54)	8.08 (0.19)
Criss 2017 [10]	Thoracoscopic	39 (1.55)	-	16 (46%)	3.3 (0.7)	31 (88.6%)	4 (11.4%)	-	-	8.33 (0.77)
	Open	38 (1.625)	-	11 (69%)	3.27 (0.4)	11 (68.8%)	5 (33.3%)	-	-	8 (1.625)
Fallahi 2017 [23]	Thoracoscopic	37.42 (8)	8 (9.2)	47 (64.4%)	2.9 (0.51)	56 (76.1%)	17 (23.9%)	-	-	-
	Open							-	-	-
Fishman 2011 [24]	Thoracoscopic	63.95 (30.18)	-	9 (75%)	4.5 (1.6)	10 (83%)	-	-	-	-
	Open	47.275 (12.02)	-	6 (66%)	3.8 (1.64)	9 (100%)	-	-	-	-
Gander 2011 [25]	Thoracoscopic	39 (1.75)	3 (5)	14 (53.8%)	3.29 (0.7)	-	-	-	7 (1.75)	8.5 (1)
	Open	38.25 (1.25)	4.75 (2.25)	8 (42.1%)	3.06 (0.54)	-	-	-	5 (2)	7.25 (1.25)
Gohda 2023 [26]	Thoracoscopic	-	742.2 (781.77)	6 (75%)	-	8(100%)	0 (0%)	-	-	-
	Open	-	210 (202.5)	3 (43%)	-	6 (86%)	1 (14%)	-	-	-
Gourlay 2009 [27]	Thoracoscopic	38.3 (1.5)	-	-	3.25 (0.61)	-	-	-	6	8
	Open	38.3 (1.65)	-	-	2.97 (0.55)	-	-	-	6	8
He 2016 [28]	Thoracoscopic	-	3.4 (1.2)	9(64%)	2.85 (0.32)	13(93%)	-	-	-	-
	Open	-	3.2 (0.6)	10(71%)	3.19 (0.64)	12(86%)	-	-	-	-
Hendrikx 2022 [29]	Thoracoscopic	36.7 (12.6)	3.3 (1.26)	-	2.4 (2.9)	3 (100%)	-	-	-	9.3 (1.26)
	Open	37.2 (4.3)	5.4 (4.5)	-	2.7 (1.02)	15 (60%)	-	-	-	7.024 (2.8)
Hosokawa 2019 [30]	Thoracoscopic	-	1 (0.8)	6 (75%)	3.02 (0.48)	7 (87%)	1 (13%)	-	-	-
	Open	-						-	-	-
Keijzer 2010 [31]	Thoracoscopic	36.5 (2)	3 (1.3)	13 (56%)	3.13 (0.67)	21 (91%)	2 (8%)	-	-	-
	Open	37.5 (2)	4.1 (2.1)	14 (60%)	3.39 (0.83)	18 (78%)	5 (21%)	-	-	-
Kim 2009 [33]	Thoracoscopic	38.5 (1.38)	-	10 (67%)	3.4 (0.69)	-	-	-	6.2 (2.07)	8.3 (1.39)
	Open	38.9 (0.519)	-		3.2 (0.17)	-	-	-	6.3 (2.07)	7.3 (2.07)
Kunisaki 2012 [34]	Thoracoscopic	-	345 (114)	-	9.1 (1.4)	-	-	-	-	-
	Open	-	759 (987)	-	9.25 (5.9)	-	-	-	-	-
Lao 2010 [35]	Thoracoscopic	35.5 (3.22)	39.5 (47.6)	12 (85%)	2.7 (0.84)	13 (92%)	1 (7%)	-	5 (1.7)	6.75 (2.14)
	Open	39 (1)	8 (5.5)	10 (58%)	3.1 (0.45)	16 (94%)	1 (5%)	-	6.25 (1.75)	7.75 (0.75)
Li 2022 [36]	Thoracoscopic	-	270 (119.1)	25 (53%)	6.55 (2.44)	38 (80%)	9 (19%)	1 (2.13%)	-	-
	Open	-	264.9 (183.3)	36 (58%)	7.04 (3.73)	51 (82%)	11 (17%)	4 (6.45%)	-	-
Lian 2024 [37]	Thoracoscopic	-	2 (4)	16(46%)	2.85 (0.61)	-	-	-	7 (3.5)	9 (2)
	Open	-	2 (69)	17(59%)	2.84 (0.74)	-	-	-	8 (4.3)	9.5 (3)
Liu 2022 [9]	Thoracoscopic	38.6 (1.6)	4.62 (2.52)	22 (59%)	3.03 (0.41)	32 (86%)	5 (13%)	-	5.43 (1.85)	8.43 (1.26)
	Open	38.4 (1.3)	4.56 (1.95)	6 (46%)	3.1 (0.25)	10 (76%)	3 (23%)	-	5.62 (1.94)	8.85 (1.14)
Mchoney 2010 [38]	Thoracoscopic	-	18 (12.8)	-	-	-	-	-	-	-
	Open	-	11.7 (18.5)	-	-	-	-	-	-	-
Muensterer 2023 [39]	Thoracoscopic	38.4 (1.6)	7.4 (3.22)	4 (80%)	3.22 (0.61)	-	-	-	-	-
	Open	37.2 (2.05)	7.7 (3.6)	4 (50%)	2.88 (0.61)	-	-	-	-	-
Okawada 2021 [40]	Thoracoscopic	-	-	20 (53%)	-	-	-	21 (57%)	4.5 (1.75)	-
	Open	-	-	102 (54%)	-	-	-	129 (70%)	5 (1.16)	-
Okazaki 2010 [41]	Thoracoscopic	37.9 (1.3)	-	5 (62%)	2.82 (0.4)	7 (87%)	1 (12%)	1 (12%)	-	-
	Open	37.5 (2)	-	6 (46%)	2.54 (0.67)	12 (92%)	1 (7%)	10 (77%)	-	-
Okazaki 2012 [42]	Thoracoscopic	-	-	4 (40%)	2.7 (0.37)	10 (100%)	-	2(20%)	-	-
	Open	-	-	6 (42%)	2.73 (0.41)	14 (100%)	-	12 (85%)	-	-
Okazaki 2015 [43]	Thoracoscopic	38 (0.87)	-	5 (33%)	2.86 (0.37)	15 (100%)	0 (0%)	12 (80%)	-	-
	Open	37 (1.45)	-	5 (50%)	2.84 (0.27)	8 (80%)	2 (20%)	10 (100%)	-	-
Qin 2019 [12]	Thoracoscopic	-	-	10 (38%)	-	-	-	-	-	-
	Open	-	-	19 (43%)	-	-	-	-	-	-
Romnek 2020 [44]	Thoracoscopic	39 (0)	-	5 (62%)	3.6 (0.89)	-	-	-	-	-
	Open	38.6 (2.39)	-	10 (50%)	3.3 (0.79)	-	-	-	-	-
Schlager 2018 [45]	Thoracoscopic	38.7 (2.5)	20 (15.4)	-	3.43 (0.52)	-	-	-	-	-
	Open	37.9 (1.6)	15.1 (13.7)	-	3.18 (0.55)	-	-	-	-	-
Shah 2023 [46]	Thoracoscopic	38 (1.6)	3.9 (1.46)	10 (62%)	3.2 (0.65)	-	-	-	-	7.2 (2.8)
	Open	37.6 (3.3)	9.3 (7.5)	9 (69%)	3.1 (0.41)	-	-	-	-	8 (1.7)
Tanaka 2013 [47]	Thoracoscopic	38.3 (1.1)	-	4 (40%)	2.88 (0.42)	10 (100%)	0 (0%)	2 (20%)	-	-
	Open	37.4 (1.6)	-	8 (57%)	2.6 (0.49)	12 (85%)	2 (0.14%)	2 (14%)	-	-
Tyson 2017 [48]	Thoracoscopic	38.75 (1.75)	7.75 (4.75)	18 (72%)	3.34 (0.54)	21 (84%)	4 (16%)	8 (33%)	-	-
	Open	39 (2.5)	7 (11)	21 (72%)	3.07 (0.64)	25 (86%)	4 (14%)	18 (67%)	-	-
Zani 2017 [49]	Thoracoscopic	38.8 (1.6)	2.6 (1.5)	13 (56%)	3.327 (0.56)	20 (87%)	3 (13%)	-	-	-
	Open	38.2 (2.6)	5 (3.7)	63 (65%)	3.115 (0.59)	84 (88%)	12 (12%)	-	-	-

*Risk of Bias Results* 

**Figure 2:**
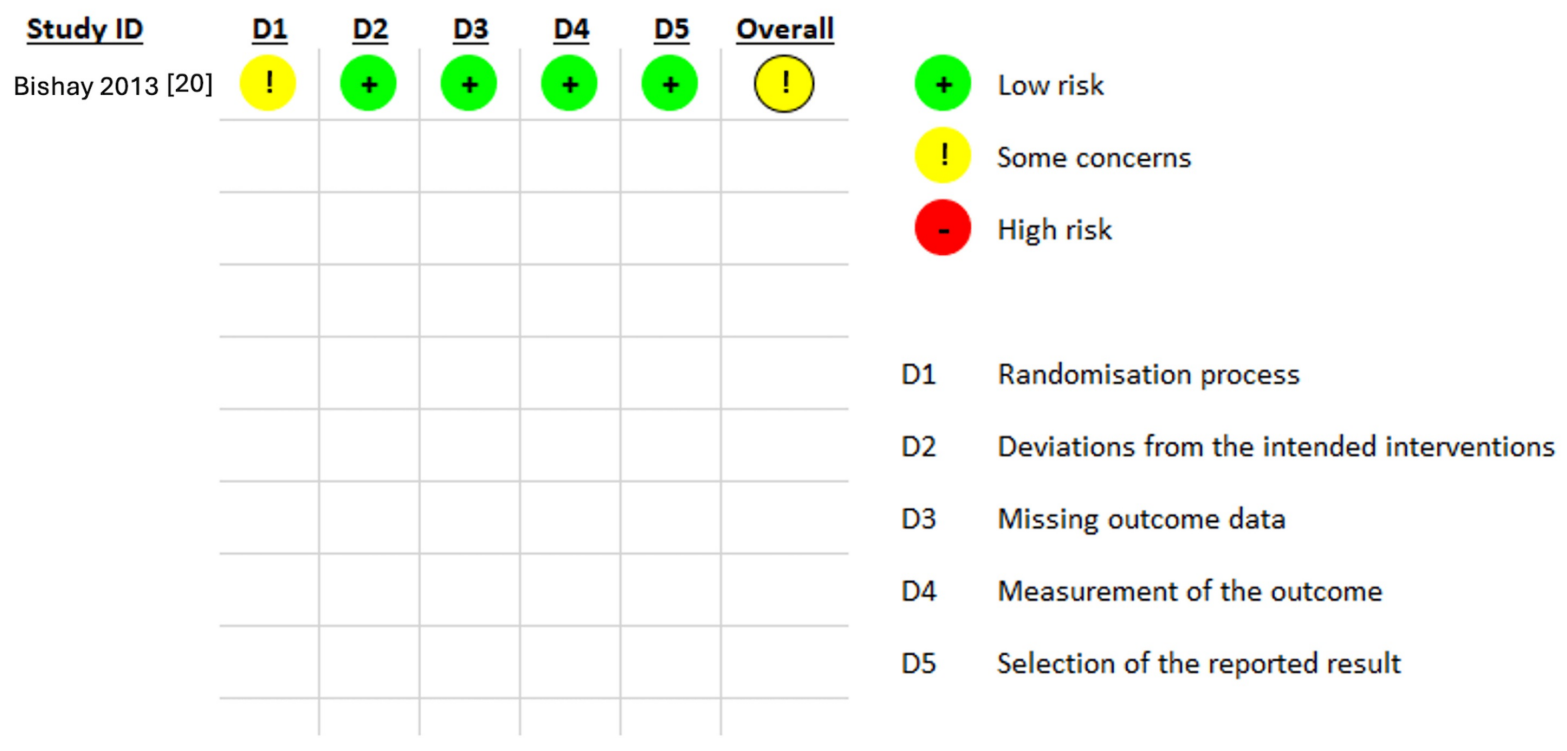
Quality assessment of the included RCT.

The quality of the retrieved observational studies was good and moderate. All studies demonstrated a good representation of the exposed cohort and proper assessment of the exposure. Tables 3 and Table 4 outline the detailed risk of bias assessment in observational studies. Only one included RCT revealed some concerns about the risk of bias due to some unexplained issues in the randomization process, as detailed in Figure 2.

**Table 3:** Quality assessment for the included cohort studies. Risk of bias in observational studies according Newcastle-Ottawa scale (NOS) tool. A study can be awarded a maximum of one star for each numbered item within the selection and exposure categories. A maximum of two stars can be given for comparability.

	Selection			Comparability	Outcome			Quality Score
	Representativeness of the exposed cohort	Selection of the non-exposed cohort	Ascertainment of exposure	Comparability of cohorts on the basis of the design or analysis	Assessment of outcome	Was follow-up long enough for outcomes to occur	Adequacy of follow-up of cohorts	
Al-iede 2015 [18]	*	*	*		*	*	*	Moderate
Bawazir 2021 [19]	*	*	*	**	*	*	*	Good
Budzanowsk 2023 [22]	*	*	*	*	*		*	Moderate
Chou 2008 [32]	*	*	*	*	*	*	*	Good
Costerus 2015 [21]	*	*	*	**	*	*	*	Good
Criss 2017 [10]	*	*	*	**	*	*	*	Good
Fallahi 2017 [23]	*	*	*		*	*	*	Moderate
Fishman 2011 [24]	*	*	*	**	*	*	*	Good
Gander 2011 [25]	*	*	*	**	*	*	*	Good
Gohda 2023 [26]	*	*	*	*	*	*	*	Good
Gourlay 2009 [27]	*	*	*	*	*	*	*	Good
He 2016 [28]	*	*	*	*	*	*	*	Good
Hendrikx 2022 [29]	*	*	*	*	*		*	Moderate
Hosokawa 2019 [30]	*	*	*		*		*	Moderate
Keijzer 2010 [31]	*	*	*	**	*	*	*	Good
Kim 2009 [33]	*	*	*	*	*	*	*	Good
Kunisaki 2012 [34]	*	*	*	*	*	*	*	Good
Lao 2010 [35]	*	*	*	*	*	*	*	Good
Li 2022 [36]	*	*	*	**	*	*	*	Good
Lian 2024 [37]	*	*	*	*	*	*	*	Good
Liu 2022 [9]	*	*	*	**	*	*	*	Good
Mchoney 2010 [38]	*	*	*	*	*	*	*	Good
Muensterer 2023 [39]	*	*	*		*	*	*	Moderate
Okawada 2021 [40]	*	*	*	*	*		*	Moderate
Okazaki 2010 [41]	*	*	*	*	*		*	Moderate
Okazaki 2012 [42]	*	*	*		*		*	Moderate
Okazaki 2015 [43]	*	*	*	**	*	*	*	Good
Romnek 2020 [44]	*	*	*	*	*		*	Moderate
Schlager 2018 [45]	*	*	*	*	*	*	*	Good
Shah 2023 [46]	*	*	*	*	*	*	*	Good
Tanaka 2013 [47]	*	*	*	*	*		*	Moderate
Tyson 2017 [48]	*	*	*	*	*	*	*	Good
Zani 2017 [49]	*	*	*	*	*	*	*	Good

**Table 4:** Quality assessment for case-control studies. Risk of bias in observational studies according to Newcastle-Ottawa scale (NOS) tool. A study can be awarded a maximum of one star for each numbered item within the selection and exposure categories. A maximum of two stars can be given for comparability.

	Selection				Comparability	Exposure			Quality score
	Is the case definition adequate?	Representativeness of the cases	Selection of controls	Definition of controls	Comparability of cases and controls based on the design or analysis	Ascertainment of exposure	The same method of ascertainment for cases and controls	Non-Response rate	
Qin 2019 [12]	*	*	*	*	*	*	*	*	Good

Study outcomes

Duration of the Hospital Stay in Days

**Figure 3:**
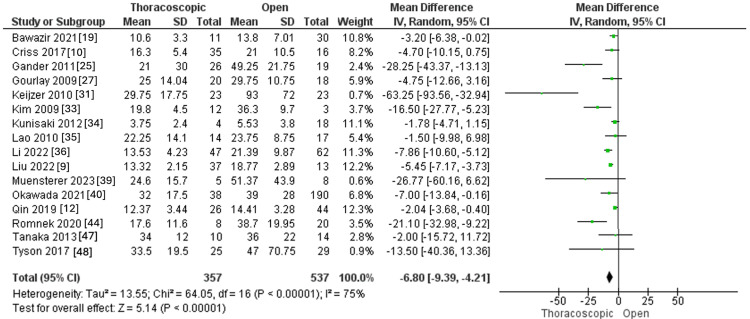
Forest plot of the duration of the hospital stay.

Seventeen studies accessed the duration of hospital stay, encompassing 357 and 537 individuals in the thoracoscopic and open groups, respectively. The pooled MD demonstrated that the thoracoscopic group was correlated with shorter hospital stay duration relative to the open group (MD=-6.80, 95 % CI [-9.39, -4.21], p<0.0001). Pooled results exhibited a significant heterogeneity (I^2^ =75%, p<0.0001), as outlined in Figure 3.

**Figure 4:**
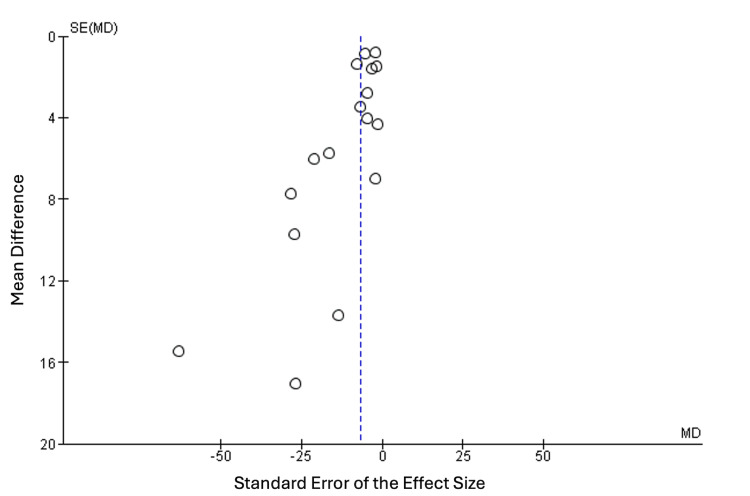
Funnel plot of the publication bias.

We constructed a funnel plot to examine the presence of any potential publication bias, and upon inspection, there was a notable symmetry around the effect estimate, indicating potential publication bias, as outlined in Figure 4.

Operation Time in Minutes

**Figure 5:**
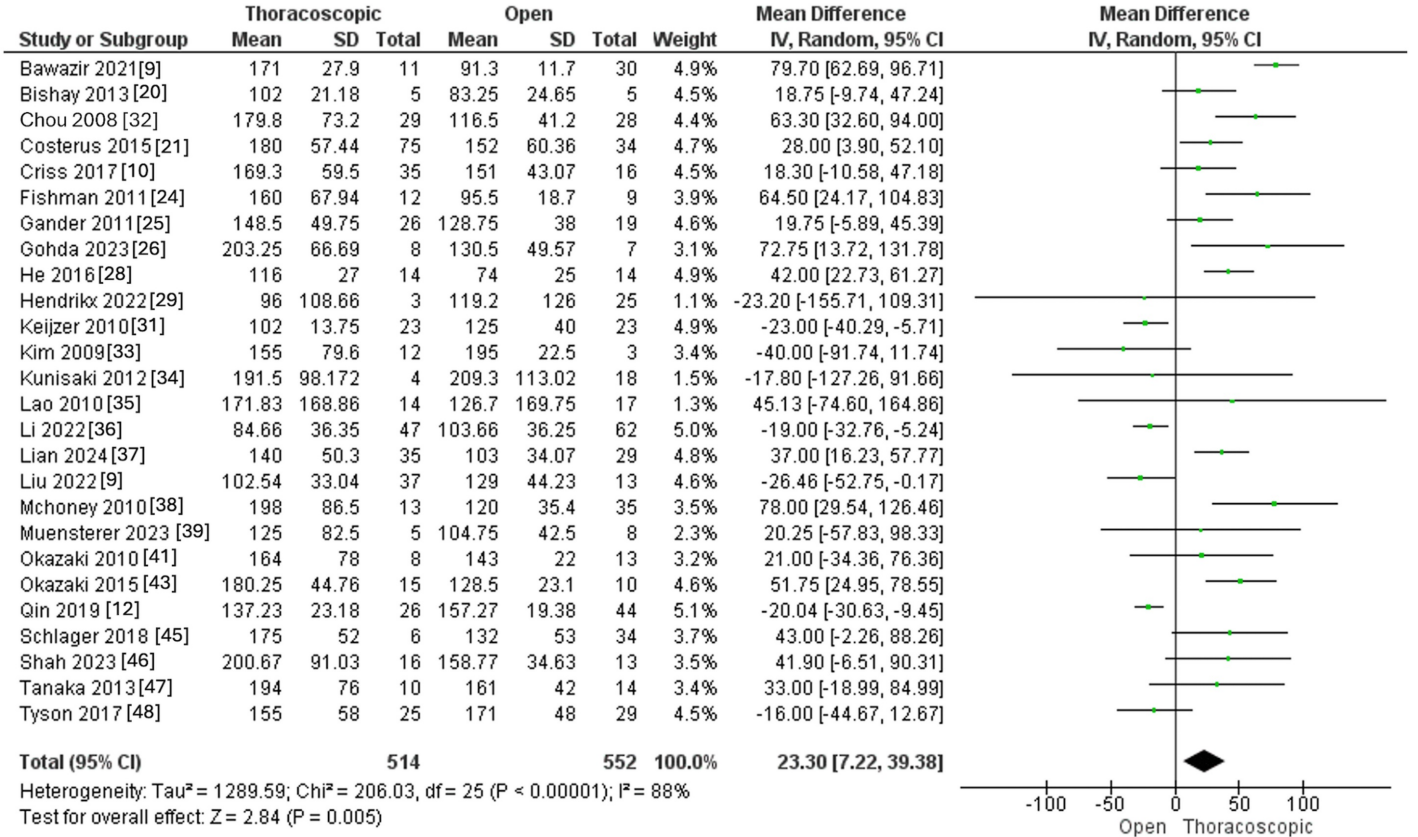
Forest plot of operation time

Operation time was measured in 26 studies that involved 514 and 552 individuals in the thoracoscopic and open groups, respectively. The pooled MD exhibited higher operating time within the thoracoscopic group than the open one (MD=23.30, 95% CI [7.22, 39.38], p=0.005). Pooled results were associated with significant heterogeneity (I^2^=88%, p<0.0001), as illustrated in Figure 5.

Intensive Care Unit Stay Duration in Days

**Figure 6:**
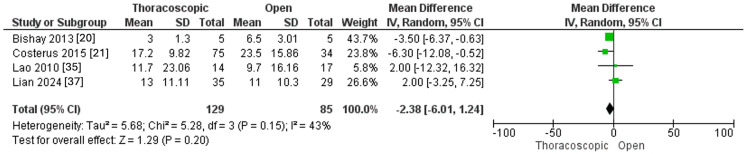
Forest plot of intensive care unit stay duration.

Four studies accessed the ICU stay, with 129 and 85 children with CDH in the thoracoscopic and open groups, respectively. There was a non-statistically substantial difference between the two procedures regarding the ICU stay duration (MD=-2.38, 95% CI [-6.01, 1.24], p=0.20). Pooled results were homogenous (I^2^ =43%, p=0.15), as illustrated in Figure 6.

Postoperative Mechanical Ventilation Duration in Days

**Figure 7:**
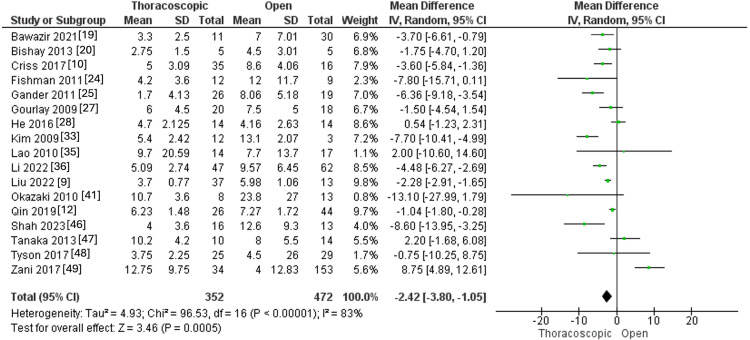
Forest plot of postoperative mechanical ventilation duration

Seventeen studies reported that the postoperative mechanical ventilation duration encompassed 352 and 472 CDH patients in the thoracoscopic and open groups. The overall MD revealed that the thoracoscopic approach was linked to a lower postoperative mechanical ventilation duration than the open approach (MD=-2.42, 95% CI [-3.80, -1.05], p=0.0005). Pooled results revealed a substantial heterogeneity (I^2^ =83%, p<0.0001), as shown in Figure 7.

Intraoperative pH

**Figure 8:**
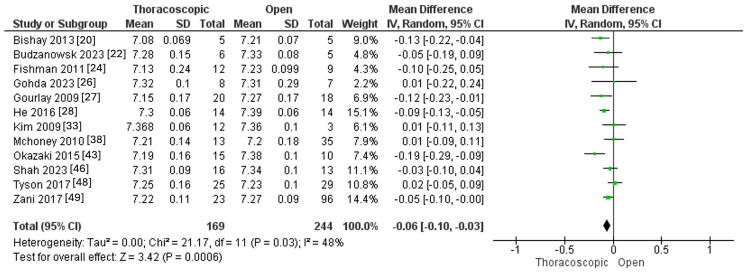
Forest plot of Intraoperative pH.

Intraoperative pH was measured in 12 studies, with 169 and 244 individuals in the thoracoscopic and open groups. The thoracoscopic procedure exhibited lower intraoperative pH than the open one (MD=-0.06, 95% CI [-0.10, -0.03], p=0.0006). Pooled results demonstrated significant heterogeneity (I^2^=48%, p=0.03), as outlined in Figure 8.

**Figure 9:**
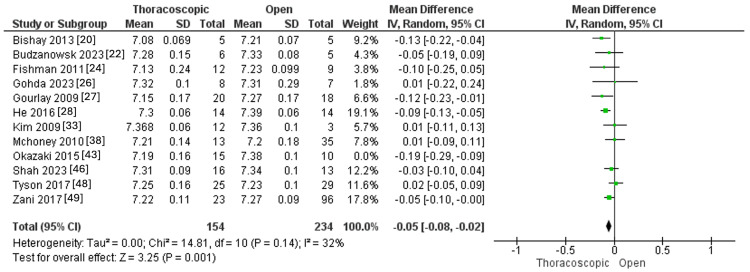
Forest plot of Intraoperative pH after excluding one study and resolving the heterogeneity.

 The observed heterogeneity was best addressed by eliminating the Okazaki 2015 et al. [43] study (I^2^ =32%, p=0.14), the meta-analysis was executed again, and the pooled effect size was not changed significantly (MD=-0.05, 95% CI [-0.08, -0.02], p=0.001), as outlined in Figure 9.

Intraoperative PCO_2_

**Figure 10:**
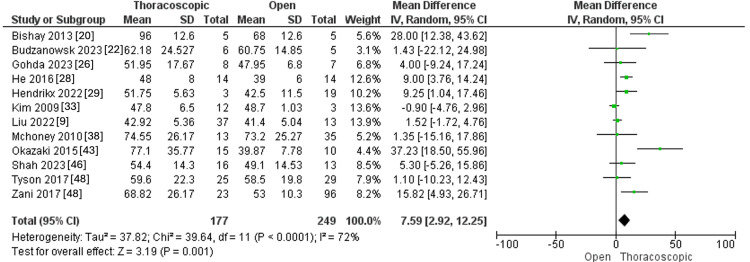
Forest plot of Intraoperative PCO2.

Intraoperative PCO_2_ was reported in 12 studies, with 177 and 249 CDH patients in the thoracoscopic and open groups, respectively. The pooled MD showed that the thoracoscopic procedure was linked to a higher intraoperative PCO2 than the open procedure (MD=7.59, 95 % CI [2.92, 12.25], p=0.001). Pooled studies demonstrated significant heterogeneity (I^2^=72%, p< 0.0001), as illustrated in Figure 10.

Mortality

**Figure 11:**
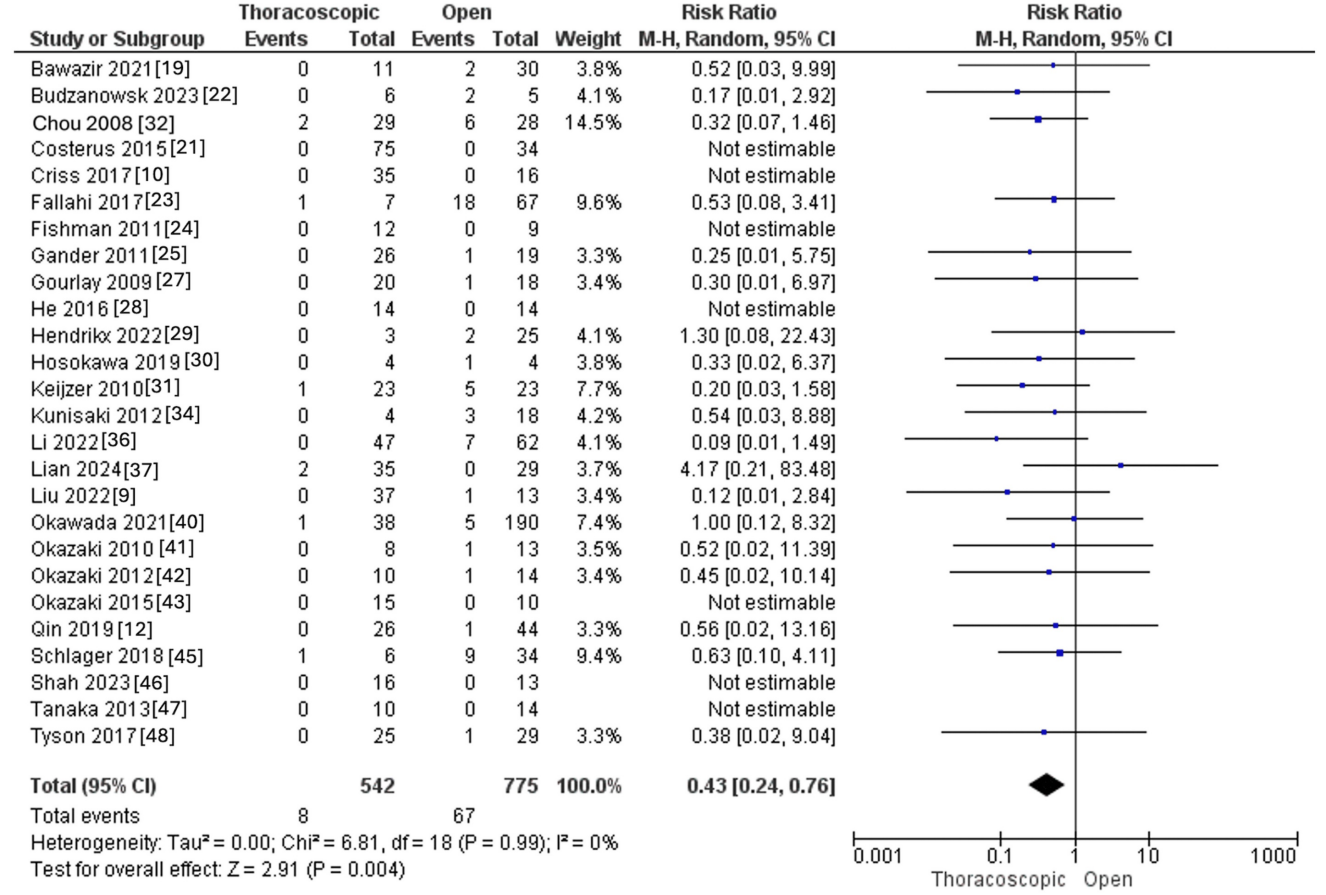
Forest plot of mortality rate.

Twenty-six studies reported the mortality, with a total of 542 and 775 children with CDH in the thoracoscopic and open groups, respectively. The pooled RR showed lower mortality rates within the thoracoscopic group than the open one (RR=0.43, 95% CI [0.24, 0.76], P=0.004). Pooled results revealed no significant heterogeneity (I^2^=0%, p=0.99), as presented in Figure 11.

Recurrence

**Figure 12:**
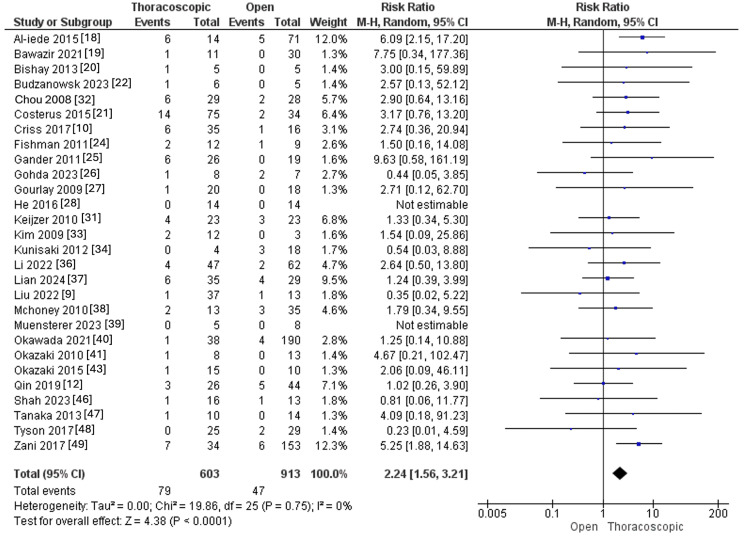
Forest plot of recurrence rate.

Recurrence was accessed in 28 studies, with 603 and 913 CDH patients in the thoracoscopic and open groups, respectively. The thoracoscopic approach revealed higher recurrence instances than the open approach (RR=2.24, 95% CI [1.56, 3.21], p=0.001). Pooled results were homogenous (I^2^=0%, p=0.75), as illustrated in Figure 12.

Pleural Effusion

**Figure 13:**
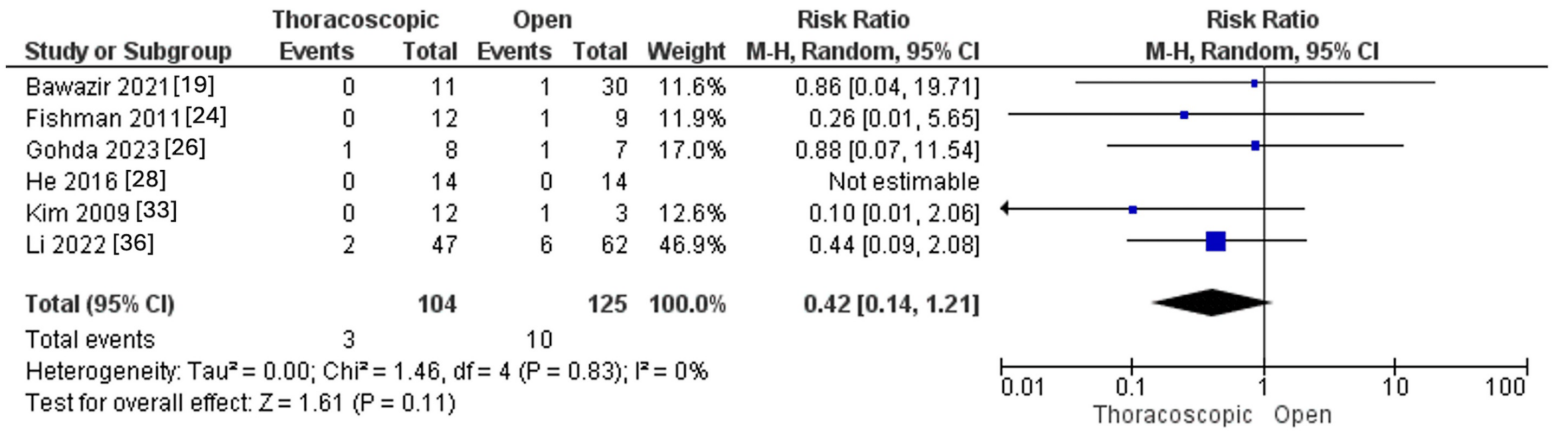
Forest plot of pleural effusion.

The thoracoscopic and open approaches revealed a similar rate of pleural effusion (RR=0.42, 95%CI [0.14, 1.21], p=0.11). Pooled studies were not heterogeneous (I^2^ =0%, p=0.83), as outlined in Figure 13.

Pneumothorax 

**Figure 14:**
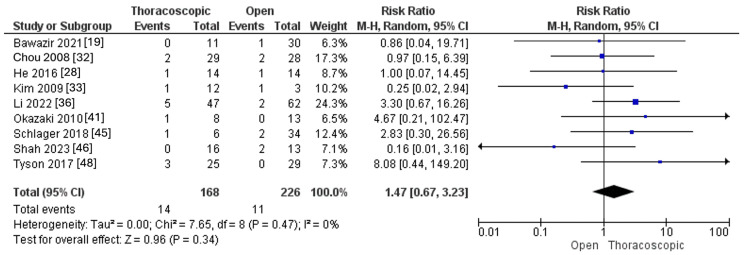
Forest plot of pneumothorax.

The pooled RR showed similar pneumothorax rates between the thoracoscopic and open groups (RR=1.47, 95% CI [0.67, 3.23], p=0.34). The pooled results showed no significant heterogeneity (I^2^=0%, p=0.47), as shown in Figure 14.

*Intestinal injury* 

**Figure 15:**
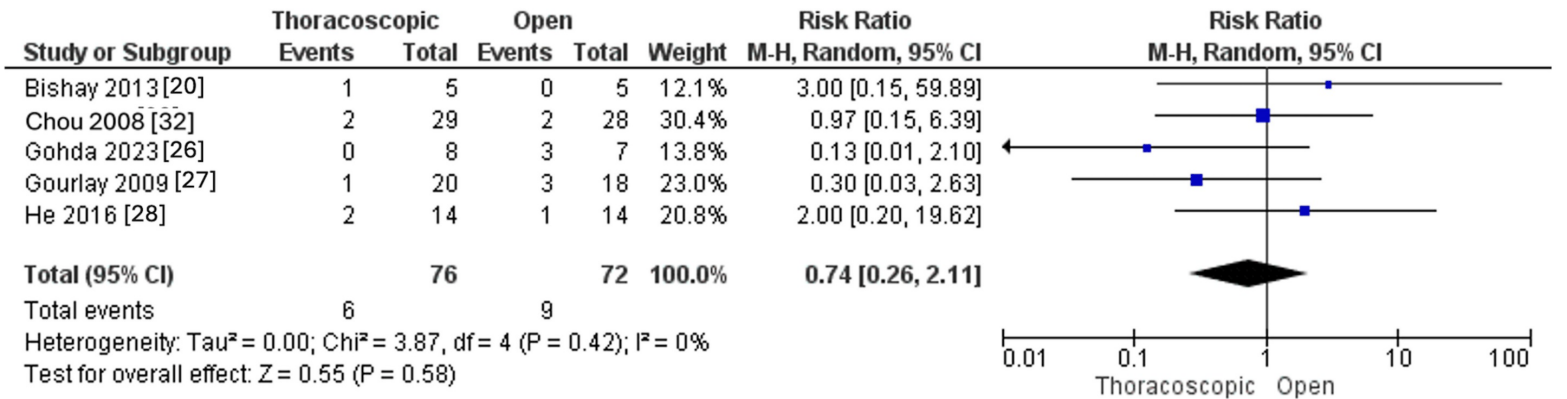
Forest plot of intestinal injury.

The pooled RR did not significantly favor any of the two approaches regarding intestinal injury (RR=0.74, 95% CI [0.26, 2.11], p=0.58). Pooled results were not heterogeneous (I^2^=0%, p=0.42), as illustrated in Figure 15.

Discussion 

Key Findings 

Our study finally involved 35 studies, with 1680 patients, comparing the thoracoscopic and open approaches for managing children with CDH. Our pooled analysis revealed that the thoracoscopic approach was significantly associated with shorter hospital stays and postoperative mechanical ventilation durations than the open approach. However, the thoracoscopic approach was linked to a higher operation time than the open one. Additionally, our primary analysis revealed lower intraoperative pH levels and higher PCO_2_ levels within the thoracoscopic group compared to the open group. Interestingly, the thoracoscopic procedure was associated with lower mortality rates when compared to the open procedure. Nevertheless, the thoracoscopic approach showed a higher recurrence risk than the open one. Finally, the pooled analysis did not favor any of the procedures regarding ICU stay, pleural effusion, pneumothorax, and intestinal injury.

Explanation of the Results

Traditionally, the open surgical approach, encompassed either thoracotomy or laparotomy, was the main surgical intervention for repairing CDH. Nevertheless, the ongoing advancements in surgical techniques have extended the implementation of minimally invasive approaches for managing individuals with CDH [50]. Thoracoscopic, one of the recently-emerged minimally invasive modalities for repairing CDH, has gained extensive adoption among pediatric surgical professionals [51]. Furthermore, the surgical outcomes for CDH repair have improved recently; however, the optimal surgical approach, either open or thoracoscopic, remains a matter of debate.

Our study aims to address this debate by pooling all the available head-to-head comparisons between thoracoscopic and open approaches. Our pooled analysis concluded that the thoracoscopic approach was associated with shorter hospital stays and lower postoperative mechanical ventilation durations than the open approach. In a previous retrospective analysis conducted by Li and his colleagues, with a total of 109 children with CDH, they found a lower hospital stay duration and postoperative mechanical ventilation duration in the thoracoscopic group compared to the open one, which aligned with our findings [36]. Nevertheless, this finding could be explained by the fact that they only utilized the thoracoscopic approach in patients with small diaphragmatic hernias, and the patients with large diaphragmatic hernias were converted to the open approach. Therefore, thoracoscopic group patients showed faster recovery than open-group patients. Qin et al. [12] conducted a retrospective observational study of 70 patients with CDH, comparing the thoracoscopic and open procedures. 

They also found that the thoracoscopic procedure was linked to a shorter hospitalization stay and lower postoperative mechanical ventilation duration than the open one. Their findings agreed with our study. However, this study was limited by its retrospective observational nature, potentially introducing some selection bias and its smaller sample size, which may cause errors in the statistical analysis. Furthermore, Kunisaki et al. [34] conducted a single-center study in 22 recurrent CDH patients. They found a non-significant difference between the two procedures regarding hospital stay duration. Their results could be attributed to their low sample size and lower hospitalization stays than other studies, eventually leading to non-significant results between the two procedures. Thoracoscopic repair is a less invasive procedure associated with a lower extent of trauma and damage compared to the open one, and it is also associated with lower postoperative complication rates compared to the open one, which could potentially explain the shorter hospital stay and lower postoperative mechanical ventilation duration observed within the thoracoscopic group [36]. 

Nevertheless, the thoracoscopic approach showed a higher operation time when compared to the open approach. He et al. [28] executed a retrospective analysis for 28 CDH patients, evaluating the thoracoscopic procedure compared to the open one. Based on their analysis, the thoracoscopic approach was associated with a higher operation time than the open one. Our findings were agreed with this study. However, this finding could be explained by the fact that the thoracoscopic group was associated with higher levels of intraoperative hypercapnia and lower pH levels, thus increasing the need for intraoperative interventions, which eventually leads to longer operation times. Moreover, this study was limited by its retrospective observational nature and small sample size. A previous retrospective study was conducted in Saudi Arabia, with 11 and 30 patients in the thoracoscopic and open groups, respectively [19]. They also found a higher operation time within the thoracoscopic approach than the open one, which aligned with our results. Extended operative durations in neonates may precipitate acidosis and hypothermia. Hence, it is recommended that the surgical risk of those individuals be evaluated and that the thoracoscopic approach be prioritized for low-risk individuals [19]. Nonetheless, Li et al. [36] and Qin et al. [12] reported a lower operation duration in the thoracoscopic group compared to the open one.

Li et al. found that the thoracoscopic group showed a lower intraoperative blood loss than the open one, resulting in shorter operative times as it reduces the need for emergent needs for interventions to control bleeding. They also utilized barbed sutures during their suturing of the diaphragm, which has subsequent benefits, such as absorbability, less bleeding, no knot response, tight sutures, and no knots during the suturing, eventually ends in lowering the operative time. Qin et al. showed that the thoracoscopic group was associated with lower incision length and more stable intraoperative hemodynamics compared to the open group, thus reducing the need for intraoperative interventions and facilitating surgical closure, thus reducing the surgery duration. The thoracoscopic approach is considered a technically complex procedure, marked by a notable learning curve, which eventually results in lowering the surgery duration over time.

Moreover, the patient's and surgery's operative characteristics could also play a role in increasing or reducing the operative time in repairing CDH individuals. Our study also revealed lower pH levels and higher PCO_2_ levels within the thoracoscopic group compared to the open one. These findings were aligned with previous studies conducted between 2013 and 2015 [20,28,43]. Previous RCT was conducted by Bishay et al. [20] to evaluate the impact of the thoracoscopic approach compared to the open one regarding the intraoperative arterial blood gases. They concluded lower pH and higher PCO_2_ levels within the thoracoscopic group than the open one. During thoracoscopic procedures, carbon dioxide (CO_2_) is insufflated into the pleural space to facilitate the surgical workspace. Still, this CO_2_ could be absorbed into the bloodstream, resulting in increased blood CO_2_ levels and hypercapnia. The increase in PCO_2_ levels could eventually lower the pH levels, resulting in acidosis [20,52]. These findings highlight the challenges in controlling intraoperative CO_2_ levels and maintaining effective ventilation during thoracoscopic procedures in infants with CDH, highlighting the necessity of effective strategies to reduce the risks of hypercapnia and acidosis. 

Our primary analysis also revealed lower mortality rates within the thoracoscopic approach than the open one. A recent multicenter retrospective study executed by Okawada and his colleagues compared the thoracoscopic and open procedures in 524 neonates with CDH [40]. They reported a higher, but not significant, incidence of mortality in the open group compared to the thoracoscopic one, 7% and 2%, respectively. This finding could be attributed to the extensive tissue trauma and higher complication rates observed within the open approach compared to the thoracoscopic one [7,8]. Furthermore, our results showed that the thoracoscopic procedure was linked to higher recurrence rates than the open procedure. In a study conducted by Zani et al. [49] comparing the two surgical approaches in a total of 119 neonates with CDH, they reported a higher recurrence rate within the thoracoscopic group compared to the open group. Interestingly, it is worthy to say that patients in the thoracoscopic group were associated with significantly higher intraoperative acidosis compared to the patients within the open group, which may explain the higher recurrence rates within the thoracoscopic procedure. In a previous retrospective study conducted in a tertiary referral center in Hong Kong, the incidence of recurrences within the thoracoscopic group (17.1%) was higher but not significant (p=0.713) compared to the open group (13.8%) [37]. However, this study was limited by its retrospective observational nature, which could introduce some selection bias, and its small sample size, which attributed to the absence of statistically significant differences between the two approaches regarding the recurrence rates. The higher recurrence rates observed within the thoracoscopic approach could be explained by its concurrent technical challenges encompassing the magnification effect within the intraoperative field, causing improper suture tension during diaphragmatic repair, and inadequate mobilization of the diaphragm rim due to restricted chest visibility [37]. Based on our pooled analysis, there was a non-significant difference between the two surgical approaches in terms of ICU stay, pleural effusion, intestinal injury, and pneumothorax. These findings could be explained by the lower number of included studies within these outcomes and the small sample size of these included studies.

Alignment with Previous Studies 

Lansdale et al. [50] performed a systematic review and meta-analysis, comparing thoracoscopic and open approaches with 143 neonates with CDH. Their findings were aligned with ours regarding the higher recurrence rates and longer operative times within the thoracoscopic group compared to the open group. However, they found no notable difference between the two procedures regarding mortality rates. They could be attributed to the few studies that fail to show any significant difference. Our study provides more powerful and updated evidence with more patients (1680) and more included studies (35).

Strengths and Limitations

Our study has multiple strength points. First, it represents the most updated and comprehensive evidence comparing the thoracoscopic and open procedures. Second, the retrieved studies were conducted in several geographical regions, which may support the generalizability of our findings to the general population. Third, the quality of our included studies ranged from good to moderate, shedding light on the good quality of the provided evidence. Nevertheless, multiple limitation points were observed in our investigation. First, many outcomes showed a notable heterogeneity among the included studies, which could be attributed to different inclusion criteria and different surgeon experiences between the included studies. Second, nearly all the study designs included were observational; only one included RCT, which raises the possibility of selecting bias.

Implication in Clinical Practice

Our results revealed substantial clinical implications for managing children with CDH. The thoracoscopic procedure was linked to shorter hospital stays and decreased postoperative mechanical ventilation durations relative to the open approach. These findings suggested potential advantages regarding patient recovery and healthcare source utilization. Nevertheless, the heightened operative durations and elevated recurrence instances with the thoracoscopic approach highlight the crucial necessity for rigorous patient selection for the optimal surgical approach. Furthermore, the higher PCO2 levels and lower intraoperative pH levels observed during the thoracoscopic procedure shed light on the importance of intraoperative monitoring of blood gas levels to reduce the risks of hypercapnia and acidosis.

Recommendations for Future Researchers

Owing to our observed limitations, heterogeneity, and the inclusion of observational studies, future research should focus on conducting large-scale- multicenter RCTS to provide stronger evidence regarding comparing the thoracoscopic and open approaches for managing neonates with CDH. Furthermore, it is essential to standardize patient inclusion criteria and surgical modalities across studies to improve the comparability of the outcomes. Finally, additional investigation into the long-term outcomes of both surgical modalities, encompassing quality of life and developmental milestones, is also warranted.

## Conclusions

Our pooled analysis revealed that the thoracoscopic approach was associated with shorter hospital stays and lower postoperative mechanical ventilation durations than the open approach. However, it also revealed longer operative time, lower pH Levels, and higher PCO_2_ levels with the thoracoscopic procedure compared to the open one. Finally, the thoracoscopic approach was linked to lower mortality instances and higher recurrence rates relative to the open approach. Further large randomized controlled trials with extended follow-up periods are still essential to support or neglect our findings.
